# Initiation response, maximized therapeutic efficacy, and post-treatment effects of biological targeted therapies in myasthenia gravis: a systematic review and network meta-analysis

**DOI:** 10.3389/fneur.2024.1479685

**Published:** 2024-10-28

**Authors:** Huahua Zhong, Zhijun Li, Xicheng Li, Zongtai Wu, Chong Yan, Sushan Luo, Chongbo Zhao

**Affiliations:** ^1^Huashan Rare Disease Center and Department of Neurology, Huashan Hospital, National Center for Neurological Disorders, Fudan University, Shanghai, China; ^2^Department of Neurology, Tongji Hospital, Tongji Medical College, Huazhong University of Science and Technology, Wuhan, China; ^3^Faculty of Biology, University of Cambridge, Cambridge, United Kingdom

**Keywords:** myasthenia gravis, neonatal fragment crystallizable receptor, complement, drug response, meta-analysis

## Abstract

**Background:**

As targeted drug development in myasthenia gravis (MG) continues to advance, it is important to compare the efficacy of these drugs for better clinical decision-making. However, due to the varied regimens and dosages used in clinical trials for different drugs, a standardized comparison between them is necessary.

**Methods:**

This study enrolled participants in phase II and III trials of innovative targeted drugs for MG. The primary outcome was the change in Quantitative Myasthenia Gravis score (MG-QMG) from baseline. The efficacy of all drugs at four time points was separately analyzed at four time points: initiation 1 week, initiation 4 weeks, maximized response, and post last dose 4 weeks. A network meta-analysis was conducted to compare the results of the different drugs.

**Results:**

A total of 9 drugs, including Efgartigimod, Rozanolixizumab, Batoclimab, Eculizumab, Belimumab, Zilucoplan, Ravulizumab, Nipocalimab, Rituximab, derived from 12 studies were analyzed. At the initiation 1-week time point, three drugs exhibited significant improvement compared to the placebo effect: Efgartigimod, Zilucoplan, Rozanolixizumab. At the initiation 4-week time point, four drugs showed significant improvement compared to the placebo effect: Efgartigimod, Rozanolixizumab, Batoclimab, Zilucoplan. At the maximized response time point, six drugs achieved significant improvement compared to the placebo effect: Efgartigimod, Rozanolixizumab, Batoclimab, Eculizumab, Zilucoplan, Ravulizumab. At the post last dose 4-week point, all drugs statistically showed no significant difference from the placebo.

**Conclusion:**

Although the MG subtypes were not consistent across trials, within the regimen design of each trial, neonatal Fc receptor inhibitors—represented by Efgartigimod, Rozanolixizumab, and Batoclimab—exhibited the most effective response rates when compared to complement and B-cell inhibitor drugs.

## 1 Introduction

Myasthenia gravis (MG) is an autoimmune disease characterized by muscle weakness, which can progress to an emergent life-threatening condition called myasthenic crisis (1). Although most patients respond to traditional immunosuppressants, around 10% of patients remain unresponsive as their symptoms persist after different treatments (2). Furthermore, the side effects of traditional immunosuppressant drugs also deter patients from taking them regularly (3). Around 18–34% of MG patients may experience relapses (4). All of the above challenges contribute to a heavy disease burden on MG patients (5).

In the recent decade, innovative biological targeted drugs have been developed to address these unmet needs. These targeted drugs are designed based on the underlying pathogenesis mechanism of MG. The pathogenic autoantibodies of MG target essential protein components of the postsynaptic membrane at the neuromuscular junction, including acetylcholine receptor (AChR), muscle-specific kinase (MuSK), and low-density lipoprotein receptor-related protein (LRP4) (6). Double seronegative MG is also observed in approximately 15% generalized MG and 50% ocular MG (7). Autoreactive B and T cells interact each other and contribute to the production of Abs, which block the AChR binding site, promote AChR internalization and degradation, or activate the classical complement pathway, leading to membrane attack complex (MAC) formation and resulting in postsynaptic membrane damage (8). These novel biological agents, including neonatal Fc receptor (FcRn) inhibitors (for antibody clearance), complement inhibitors (inhibiting MAC formation at the neuromuscular junctions), and B cell inhibitors (reducing autoantibody-producing B cells), have been developed to target these mechanisms. However, there is currently no head-to-head comparison study of these agents.

In the completed randomized controlled trial studies so far, changes in MG Activities of Daily Living (MG-ADL) or Quantitative Myasthenia Gravis (MG-QMG) relative to baseline have been adopted as either primary or secondary study endpoints, rendering them relatively comparable. Several meta-analyses have been conducted to compare the efficacy of these innovative drugs (9–12). While similar safety results were achieved, different conclusions were made regarding efficacy. We believe this could be explained by the variations in the clinical trial data and the different regimens used in these trials. For example, some drugs were administered persistently during the trial [e.g., Eculizumab (13)], while others were stopped several months before the trial ended [e.g., Belimumab (14)]. Hence, it would be difficult to draw conclusions when only efficacy at the end of trials was compared. Additionally, different dosage groups made it imprecise to consider the efficacy of all groups as representative of one drug [e.g., Zilucoplan (15)].

We conducted a meta-analysis to address the issues related to the innovative targeted drugs used in MG treatment. Our analysis focused on the common time points in the regimen design of each trial, investigating the initial response, maximized therapeutic efficacy, and post-treatment effects of these drugs.

## 2 Methods

### 2.1 Search strategy

**Figure 1:**
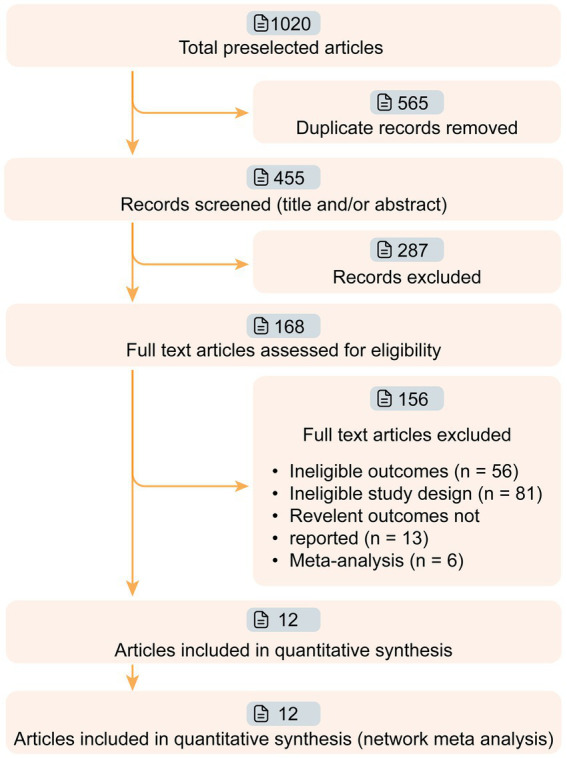
Workflow of article screening. Twelve articles of phase II/III targeted drugs for myasthenia gravis (MG) were screened from 1,020 relevant articles. The efficacies of nine drugs (Efgartigimod, Nipocalimab, Eculizumab, Ravulizumab, Belimumab, Rituximab, Rozanolixizumab, Batoclimab, and Zilucoplan) were compared in this network meta-analysis.

We conducted a thorough search of several databases including PubMed, Web of Science, the Cochrane Central Register of Controlled Trials (CENTRAL), and ClinicalTrials.gov to identify randomized controlled trials of targeted therapies for MG. The search was carried out from the inception of databases to January 20th, 2024 (Figure 1). A combination of search strings was used to filter related studies, including ‘myasthenia’ combined with ‘blind’ or ‘randomized’ [“myasthenia” AND (‟blind” OR ‟randomized”)]. To ensure accuracy, three reviewers (HZ, ZL and XL) searched and screened the eligible studies independently, and any inconsistencies were resolved by consulting a fourth reviewer (SL). Additionally, the references for the included full-text articles and relevant systematic reviews were also screened.

### 2.2 Eligibility criteria

We conducted a meta-analysis of targeted drug trials in MG using a common two-stage study design (treatment-observation) (16, 17). Treatments are usually continuously administered in the treatment stage, and a no-drug observation stage often follows to evaluate sustainability. We based our analysis on four shared time points: (i) 1 week after the first administration (initiation 1w), (ii) 4 weeks after the first administration (initiation 4w), (iii) optimal efficacy of the targeted drug group during the treatment stage (maximized response), and (iv) 4 weeks after the last dose of treatment at the observation stage (post last dose 4w). We screened studies containing any of the above data.

The following inclusion criteria were used to screen those targeted drug trials in MG: (i) phase II or III double-blind and randomized controlled trials, (ii) inclusion of MG-QMG score change from baseline as an outcome variable, and (iii) clinical trials of innovative targeted drugs compared to placebo. Exclusion criteria included: (i) studies not written in English; (ii) reviews, observational studies, case reports, and conference abstracts.

### 2.3 Outcome definition and data extraction

The primary outcome defined in this study is the change in MG-QMG score from the baseline. Since MG-QMG change was compared in multiple timepoints, no further secondary endpoints are needed. The MG-QMG scale was introduced in 1988 and has become one of the most widely used scales in MG clinical trials. Unlike the patient-reported MG-ADL scale, MG-QMG is a 13-item direct physician assessment scoring system that quantifies disease severity based on the impairments of body functions and structures (18). The total QMG score ranges from 0 to 39, where higher scores indicate greater disease severity. In addition, this scale assesses muscle strength and fatigability using objective measures of double vision, ptosis, facial muscles, dysphagia, and limb weakness (19). Data extracted from each study included the first author, publication year, trial phase, subjects, regimen, sample size, age, sex, and antibody type. Data extraction was independently performed by three reviewers (HZ, ZL and XL). Any conflicts during data extraction were resolved by consensus.

In studies where the primary outcome was not directly reported, a figure data extraction tool, DigXY,^1^ was utilized to digitize the mean and standard deviation (SD) from the figure. In studies where the 95% confidence interval was provided instead of SD, we inferred SD using the method recommended by Cochrane (20). To perform this network meta-analysis the following three assumptions must be satisfied: similarity or homogeneity assumption generally applies to direct comparisons, transitivity assumption applies to indirect comparisons, and consistency assumption applies to mixed comparisons (21).

### 2.4 Statistical analysis

This systematic review and network meta-analysis were reported according to the Preferred Reporting Items for Systematic Reviews and Meta-Analyses (PRISMA) guidelines. We used the Cochrane Risk of Bias tool version 2.0^2^ to assess the risk of bias in the included studies, including randomization, allocation concealment, blinding, missing outcome data, and selective reporting of results. Three reviewers independently completed the bias risk assessment (HZ, ZL, and XL), and any differences were resolved by consensus.

The efficacy (standardized mean differences) of each targeted drug compared to placebo at different time points was pooled using the random-effects model, and heterogeneity was calculated using the restricted maximum likelihood (REML) approach. Heterogeneity across trials was calculated using the Cochrane Q test, and I2 values were reported. All data and figures were analyzed and generated using R (version 4.3.7). R packages, including tidyverse, rstatix, meta, netmeta, dmetar, and robvis, were used.

## 3 Results

### 3.1 Study characteristics

We retrieved 1,020 articles and assessed 168 full-text articles after article screening (Figure 1). Twelve eligible articles (treatment *n* = 543, and placebo *n* = 517) were enrolled in the final network meta-analysis (Table 1). Among them, five were phase II and seven were phase III. In total, nine targeted drugs were compared in this network meta-analysis, of which six were administered intravenously (Efgartigimod, Nipocalimab, Eculizumab, Ravulizumab, Belimumab, Rituximab) (13, 14, 22–25) and three were administered subcutaneously (Rozanolixizumab, Batoclimab, and Zilucoplan) (15, 26–30). No ethical concerns were raised for any of these articles. Although different dosing regimens (5 for once weekly, 2 for once every 2 weeks, 2 for daily, and 1 for single-infusion) were applied in each trial, seven studies adopted a complete two-stage study design (treatment-observation), with an observation period ranging from 4 to 48 weeks. Most studies (10 out of 12) were conducted with generalized MG patients, while two studies were specifically conducted in refractory (Eculizumab) and newly-onset (Rituximab) generalized MG patients.

**Table 1:** Characteristics of enrolled clinical trials.

Reference	Interventions	Drug type	Study Phase	Subject	Regiment	Sample size (I/C)	Mean age, (I/C, years)	Female (I/C, %)	AChR+ Patients (I/C, %)	Baseline QMG score (I/C)	Initiation 1w Response (QMG change, I/C (weeks))	Initiation 4w Response (QMG change, I/C (weeks))	Maximized Response (QMG change, I/C (weeks))	Observation 4w Response (QMG change, I/C (weeks))
Howard et al. (22)	Efgartigimod	FcRn	Phase 3	Generalized MG (MG-ADL ≥ 5)	IV Efgartigimod, administered 10 mg/kg QW for 4 weeks one cycle, repeated as needed, no sooner than 8 weeks after initiation of the previous cycle.	84/83	45.9 ± 14.4/48.2 ± 15.0	75%/66.3%	77.4%/77.1%	16.2 ± 5.0/15.5 ± 4.6	−2.8 ± 4.2/0.0 ± 2.8 (1w)	−6.2 ± 6.0/−0.9 ± 3.4 (4w)	−6.3 ± 5.6/−1.0 ± 4.2 (5w)	−2.9 ± 4.7/−1.2 ± 3.1 (8w)
Antozzi et al. (23)	Nipocalimab	FcRn	Phase 2	Generalized MG (MG-QMG ≥ 12)	IV Nipocalimab, administered 60 mg/kg Q2W for 8 weeks, followed by 8 weeks of observation.	13/14	63.0 (27.0–76.0)/60.5 (25.0–83.0)	35.7%/57.1%	92.9%/92.9%	16.9 ± 2.8/17.6 ± 4.2	−2.8 ± 3.7/−2.1 ± 3.3 (1w)	−4.0 ± 4.4/−2.4 ± 2.3 (4w)	−5.9 ± 5.2/−3.9 ± 3.1 (8w)	−5.1 ± 3.3/−4.0 ± 3.0 (12w)
Bril et al. (26)	Rozanolixizumab	FcRn	Phase 3	Generalized MG (MG-QMG ≥ 11)	SQ Rozanolixizumab, administered 10 mg/kg QW for 6 weeks, followed by 8 weeks of observation.	67/67	51.9 ± 16.5/50.4 ± 17.7	52.2%/70.1%	89.6%/88.1%	15.6 ± 3.6/15.8 ± 3.5	−2.6 ± 1.8/−0.8 ± 2.0 (1w)	−5.1 ± 4.9/−1.2 ± 3.5 (4w)	−5.6 ± 5.4/−0.9 ± 4.2 (6w)	−2.6 ± 4.8/−1.4 ± 4.6 (10w)
Yan et al. (27)	Batoclimab	FcRn	Phase 2	Generalized MG (MG-QMG ≥ 6)	SQ Batoclimab, administered 680 mg/kg QW for 5 weeks in double-blinded period, and 340 mg/kg Q2W for 6 more weeks, and then followed up for 6 weeks without treatment.	11/9	40.6 ± 16.8/40.2 ± 9.3	81.8%/77.8%	100.0%/88.9%	18.8 ± 6.1/14.9 ± 5.0	−3.4 ± 2.5/−1.0 ± 3.7 (1w)	−7.7 ± 5.4/−1.4 ± 2.0 (4w)	−8.0 ± 4.6/−1.7 ± 3.6 (6w)	−4.5 ± 4.0/−5.3 ± 5.5 (15w)
Nowak et al. (29)	Batoclimab	FcRn	Phase 2	Generalized MG (MG-QMG ≥ 12)	SQ Batoclimab, administered 680 mg/kg QW for 6 weeks, and followed by 340 mg/kg Q2W of open-label extension for 6 weeks.	6/6	70.8 ± 14.2/41.0 ± 15.4	16.7%/66.7%	100%/100%	16.2 ± 2.2/17.2 ± 3.2	−1.7 ± 3.1/−3.3 ± 2.0 (1w)	−3.2 ± 3.7/−2.5 ± 2.9 (4w)	−4.3 ± 3.7/−2.9 ± 3.3 (5w)	\
Yan et al. (28)	Batoclimab	FcRn	Phase 3	Generalized MG (MG-QMG ≥ 11)	SQ Batoclimab, administered 680 mg/kg QW for 6 weeks, followed by 4 weeks of observation without treatment.	67/64	43.8 ± 13.9/43.7 ± 13.5	59.7%/75.0%	97.0%/92.2%	17.9 ± 4.8/18.3 ± 4.9	−2.0 ± 2.9/−0.9 ± 2.9 (1w)	−5.4 ± 3.7/−1.7 ± 3.7 (4w)	−6.4 ± 4.0/−1.6 ± 4.0 (6w)	−3.6 ± 4.0/−1.6 ± 4.1 (9w)
Howard et al. (13)	Eculizumab	Complement	Phase 3	Refractory generalized MG (MG-ADL ≥ 6)	IV Eculizumab, administered 900 mg on day 1 and weeks 1, 2, and 3; 1,200 mg at week 4; maintenance dosing 1,200 mg every second week thereafter until week 26.	62/63	38 ± 17.8/38.1 ± 19.6	66.1%/65.1%	100%/100%	17.3 ± 5.1/16.9 ± 5.6	−1.9 ± 3.3/−0.9 ± 3.3 (1w)	−3.3 ± 4.4/−1.5 ± 4.4 (4w)	−4.6 ± 4.8/−1.6 ± 4.6 (26w)	\
Howard et al. (15)	Zilucoplan	Complement	Phase 2	Generalized MG (MG-QMG ≥ 12)	SQ Zilucoplan, administered 0.3 mg/kg QD for 12 weeks.	14/15	54.6 ± 15.5/48.4 ± 15.7	28.6%/84.3%	100%/100%	19.1 ± 5.1/18.7 ± 4.0	−3.9 ± 3.8/−2.2 ± 3.8 (1w)	−5.4 ± 4.6/−3.7 ± 4.3 (4w)	−6.3 ± 4.5/−3.5 ± 4.6 (12w)	\
Howard et al. (30)	Zilucoplan	Complement	Phase 3	Generalized MG (MG-QMG ≥ 12)	SQ Zilucoplan, administered 0.3 mg/kg QD for 12 weeks.	88/86	52.6 ± 14.6/53.3 ± 15.7	60.5%/53.4%	100%/100%	18.7 ± 3.6/19.4 ± 4.5	−3.8 ± 3.6/−1.6 ± 3.4 (1w)	−5.7 ± 4.5/−2.8 ± 4.8 (4w)	−6.2 ± 7.6/−3.3 ± 5.1 (12w)	\
Vu et al. (50)	Ravulizumab	Complement	Phase 3	Generalized MG (MG-ADL ≥ 6)	IV Ravulizumab, administered >2,400 mg on day 1, day 15, and every 8 weeks thereafter for 26 weeks	86/89	58.0 ± 13.8/53.3 ± 16.1	51.2%/50.6%	100%/100%	14.8 ± 5.2/14.5 ± 5.3	−1.7 ± 3.1/−0.5 ± 3.4 (4w)	−2.6 ± 3.8/−0.8 ± 3.9 (4w)	−3.2 ± 4.4/−1.1 ± 4.3 (18w)	\
Hewett et al. (14)	Belimumab	B cell	Phase 2	Generalized MG (MG-QMG ≥ 8)	IV Belimumab, administered 10 mg/kg throughout the 24-week treatment phase (weeks 0, 2, 4, 8, 12, 16, and 20) and followed by 12-week observation period.	18/21	52.7 ± 17.3/59.0 ± 13.9	55.6%/66.7%	100%/88.9%	12.0 (8.0–19.5)/12.5 (6.5–23.0)	\	−1.0 ± 3.5/−1.0 ± 3.4 (4w)	−4.7 ± 4.5/−1.8 ± 4.8 (20w)	−4.2 ± 5.0/−2.3 ± 5.3 (24w)
Piehl et al. (25)	Rituximab	B cell	Phase 3	New-onset generalized MG (MG-QMG ≥ 6)	IV Rituximab, administered 500 mg of single infusion on day 1 and followed by 48 weeks	25/22	67.4 ± 13.4/58.0 ± 18.6	28.0%/31.8%	92.0%/100%	9.4 ± 4.5/9.3 ± 4.2	\	\	−6.7 ± 5.7/−5.7 ± 4.4 (48w)	\

### 3.2 Pooled efficacy results

**Figure 2:**
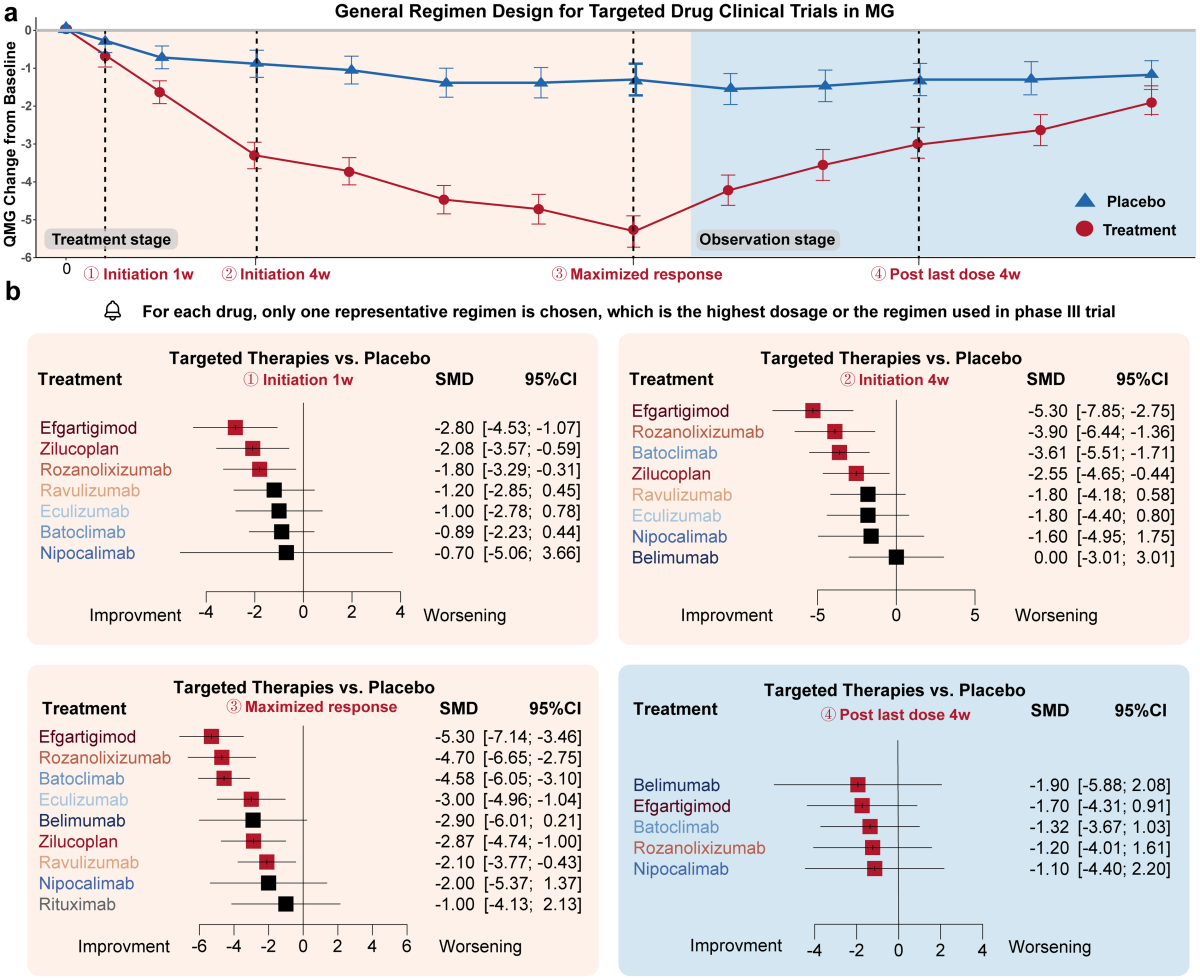
Drug efficacy comparison of different targeted drugs. **(a)** General trial design (treatment-observation two-stage regimen) of most targeted therapies in MG. Four time points were specifically selected to evaluate the initiation response, maximized therapeutic efficacy, and post-treatment effects of these drugs: initiation 1w (1 week), initiation 4w, maximized response, post-treatment 4w. **(b)** For the initiation 1w point, Efgartigimod, Zilucoplan, and Rozanolixizumab exhibited significant improvement compared to the placebo’s efficacy. For initiation 4w point, Efgartigimod, Rozanolixizumab, Batoclimab, Zilucoplan, exhibited significant improvement compared to placebo’s efficacy. For the maximized response point, Efgartigimod, Rozanolixizumab, Batoclimab, Eculizumab, Zilucoplan, and Ravulizumab exhibited significant improvement compared to placebo’s efficacy. For the post-treatment 4w point, none of the drugs exhibited a difference compared to the placebo group.

Drug efficacy in terms of MG-QMG change from baseline was compared at four time points (Figure 2):

- Initiation 1w: Three drugs exhibited significant improvement compared to the placebo effect: Efgartigimod (−2.80 [−4.54, −1.07]), Zilucoplan (−2.08 [−3.57, −0.59]), and Rozanolixizumab (−1.80 [−3.29, −0.31]).
- Initiation 4w: Four drugs exhibited significant improvement compared to the placebo effect: Efgartigimod (−5.30 [−7.85, −2.75]), Rozanolixizumab (−3.90 [−6.44, −1.36]), Batoclimab (−3.61 [−5.51, −1.71]), Zilucoplan (−2.55 [−4.65, −0.44]).
- Maximized response: Six drugs achieved significant improvement compared to the placebo effect: Efgartigimod (−5.30 [−7.14, −3.46]), Rozanolixizumab (−4.70 [−6.65, −2.75]), Batoclimab (−4.58 [−6.05, −3.10]), Eculizumab (−3.00 [−4.96, −1.04]), Zilucoplan (−2.87 [−4.74, −1.00]), Ravulizumab (−2.10 [−3.77, −0.43]). The average time needed to achieve the maximized response was 14.3 ± 12.6 weeks.
- Post last dose 4w: All drugs showed no significant difference with placebo.

In summary, Efgartigimod, Rozanolixizumab, and Zilucoplan demonstrated the fastest onset of efficacy regarding the initiation response. Efgartigimod, Rozanolixizumab, and Batoclimab were the most effective drugs in terms of maximized response. However, none of the targeted drugs maintained efficacy after administration was discontinued for 4 weeks. Overall, FcRn inhibitors, represented by Efgartigimod, Rozanolixizumab, and Batoclimab, exhibited better responses compared to complement and B-cell inhibitor drugs, which may provide as an effective fast relieve therapy for those worsening patients. Overall, FcRn inhibitors, represented by Efgartigimod, Rozanolixizumab, and Batoclimab, exhibited better responses compared to complement and B-cell inhibitor drugs, which may provide an effective fast relief therapy for worsening patients.

### 3.3 Subgroup analysis

**Figure 3:**
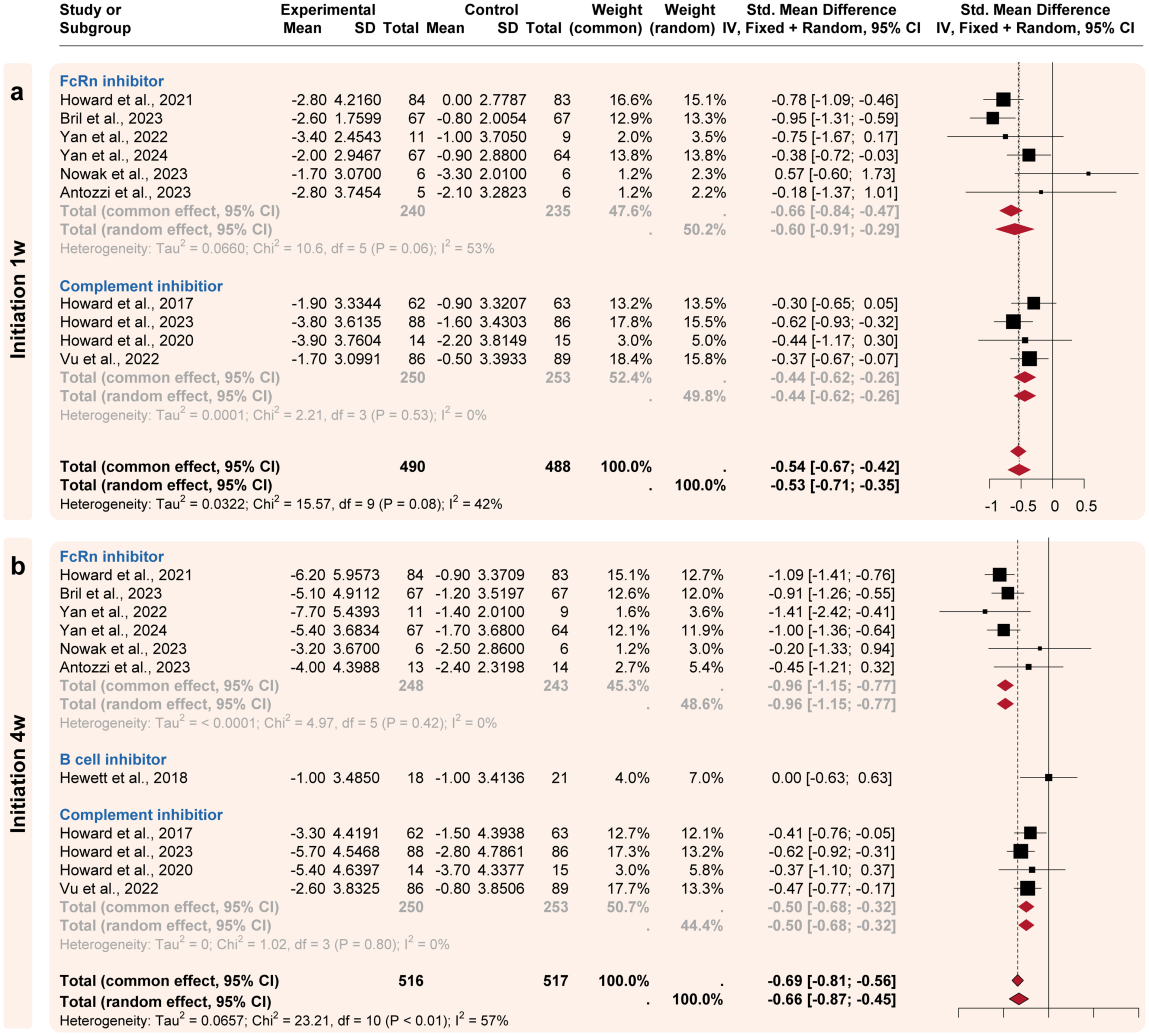
Subgroup analysis by the drug categories (initial responses). The efficacies of Neonatal Fc receptor (FcRn), complement, and B-cell inhibitors were compared at two initiation time points. **(a)** For the initiation 1w point, FcRn and complement inhibitors exhibited significant improvement compared to the placebo’s efficacy. **(b)** For the initiation 4w point, FcRn and complement inhibitors exhibited more improvement compared to the placebo’s efficacy.

**Figure 4:**
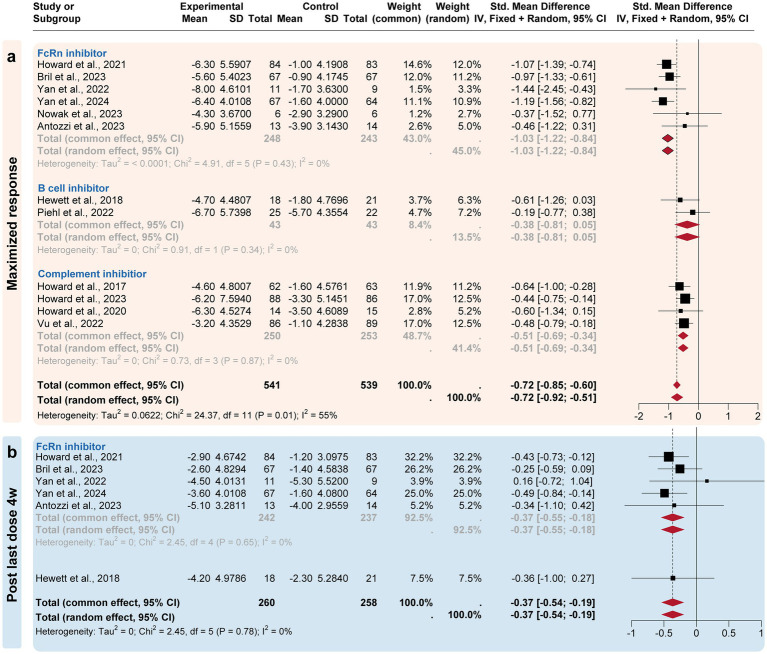
Subgroup analysis by the drug categories (maximized and post-treatment responses). The efficacies of Neonatal Fc receptor (FcRn), complement, and B-cell inhibitors were compared at three time points. **(a)** For the maximized response point, FcRn and complement inhibitors exhibited the most improvement. **(b)** For the post last dose 4w point, only FcRn inhibitors exhibited a difference compared to the placebo group.

A subgroup analysis compared the efficacy of three targeted drug categories, including FcRn, complement, and B-cell inhibitors (Figures 3, 4). FcRn inhibitor drugs (Efgartigimod, Rozanolixizumab, Batoclimab, and Nipocalimab) consistently exhibited the most significant efficacy compared to placebo: −0.66 [−0.91, −0.29] at the initiation 1-week time point, −0.96 [−1.15, −0.77] at the initiation 4-week time point, −1.03 [−1.22, −0.84] at the maximized response timepoint, and − 0.37 [−0.55, −0.18] at the post last dose 4-week time point. On the other hand, complement inhibitor drugs (Eculizumab, Zilucoplan, and Ravulizumab) generally showed suboptimal efficacy than placebo at the initiation1-week mark (−0.44 [−0.62, 0.26]), the initiation 4-week mark (−0.51 [−0.73, 0.29]) and maximized response time point (−0.53 [−0.75, −0.31]). B-cell inhibitor drugs (Belimumab and Rituximab) exhibited no significant difference compared to placebo over a time range of 24–48 weeks. In summary, the analysis showed that FcRn inhibitors were the most efficient treatment for MG in terms of both initial and maximized response, with potential efficacy extending to the 4-week post-treatment maintenance.

### 3.4 Publication bias detection, heterogeneity, and sensitivity analysis

**Figure 5:**
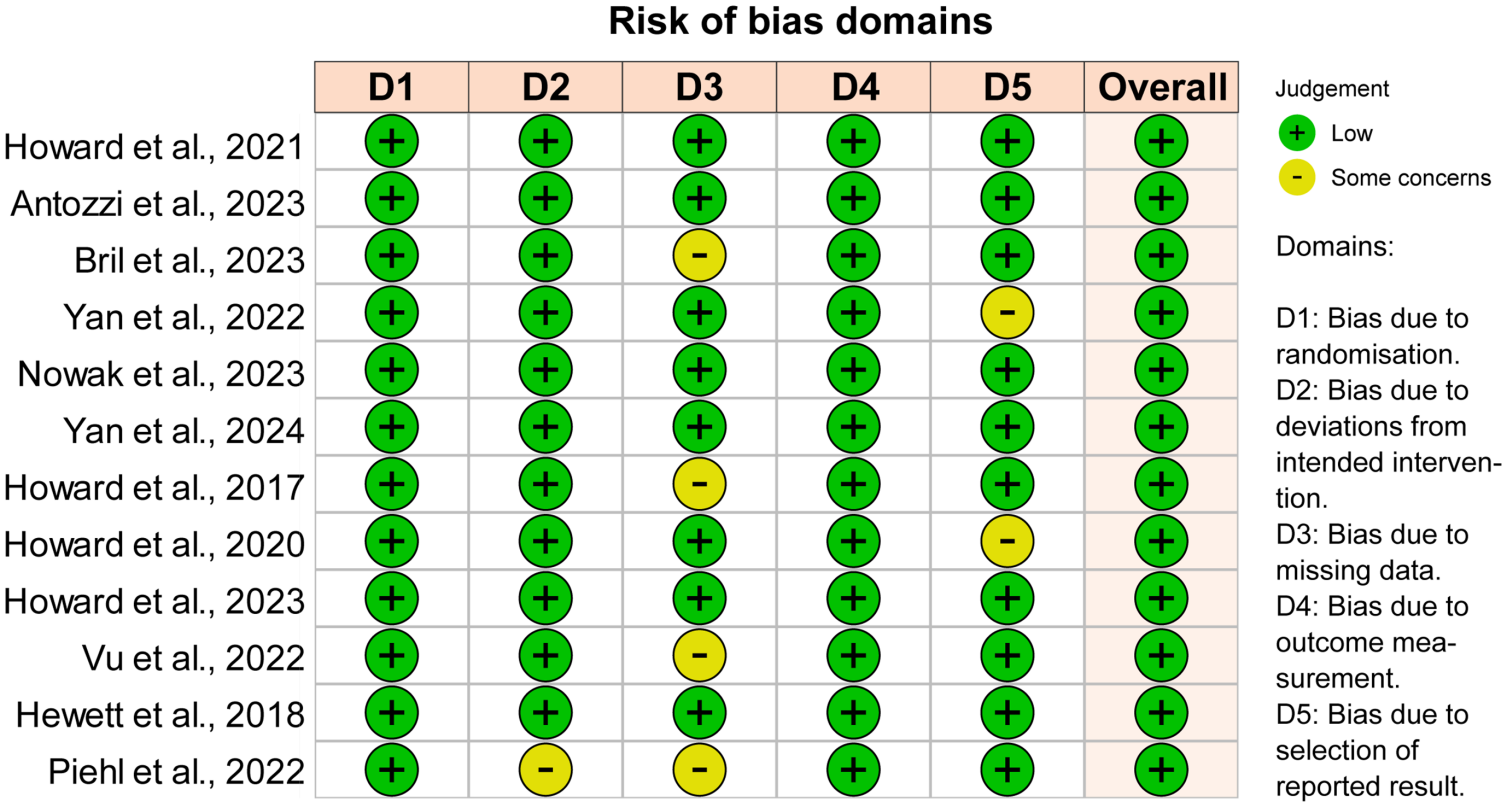
Quality and bias evaluation of enrolled studies. The overall risk of bias for all 12 enrolled studies is “Low”.

The overall quality of the studies included in the analysis was high because these studies were well-designed randomized clinical trials. Seven instances of ‘Some concerns’ (11.7%) were raised in 60 domains across the total of 12 publications (Figure 5). The heterogeneity I2 values at the four time points were 56.0% at initiation 1w, 37.0% at initiation 4w, 12.3% at maximized response, and 35.7% at post-treatment 4w. To reduce heterogeneity and potential biases, a sensitivity analysis including only phase III trials was conducted (Supplementary material 1). Efgartigimod, Rozanolixizumab, and Batoclimab remained the most effective drugs in the network comparison of seven phase III trials. Similarly, FcRn inhibitors consistently exhibited a better response than complement and B-cell inhibitors.

## 4 Discussion

Traditional immunosuppressive treatments for MG include corticosteroids, azathioprine, tacrolimus, mycophenolate, cyclophosphamide and ciclosporin. However, there was a lack of high-quality and evidence-based studies supporting the use of these drugs. Previous randomized controlled trials comparing corticosteroids, azathioprine, and tacrolimus with placebos did not yield significantly positive results (31–34). Despite this, these drugs are widely used in the real world. They are usually associated with significant side effects, and some patients are refractory to these drugs (35).

In recent years, there has been a surge in the development of innovative biological targeted drugs to meet the unmet needs of MG. These drugs are designed to target specific molecules, such as antibodies, complement, and B cells, offering a more personalized approach to treatment. While these drugs have already been proven to be effective and safe in previous phase III clinical trials and meta-analyses (9–12), more detailed analysis regarding different regimens is needed to help physicians choose the best drug for suitable conditions and treatment goals. However, there is no head-to-head study directly comparing the efficacy and safety of these novel biologic targeted drugs in treating MG. Inspired by the several previous trial designs for MG, this study aimed to compare drug efficacy within the same time points, minimizing the biases caused by different regimens and dosages as much as possible.

Direct clearance of pathogenic antibodies is one of the most important treatment strategies for MG, as demonstrated by the favorable outcomes of IV immunoglobulin (IVIg), plasma exchange (PLEX) and immunoadsorption in moderate to severe MG patients (36). Various antibody clones in AChR-MG patients may cause various pathogenic effects, and single autoreactive clones can mediate multiple modes of pathology (37). Complement activation occurs downstream of pathogenic IgG1 antibody subtypes of AChR or LRP4. Studies have shown that in MG patients and animal models, an active complement system is primarily responsible for the development of muscle weakness. Mice deficient in intrinsic complement regulatory proteins exhibited a significant increase in the destruction of the neuromuscular junction (38). The differences in targets determine the suitability of FcRn for various antibody-mediated MG, while complement inhibitors are mainly confined to AChR-MG.

As an endogenous IgG antibody-clearing method, FcRn inhibitors are more selective and durable than PLEX, and more effective than IVIg (39). Consequently, the reduction of antibody levels (≈75%) achieved by FcRn inhibitors resembles that of plasmapheresis (PLEX) (40). In this study, FcRn inhibitors exhibit a better response rate and greater efficacy than complement inhibitors, followed by B-cell inhibitors. Among the three FcRn inhibitors studied (Efgartigimod, Rozanolixizumab, and Batoclimab), the most effective initial and maximized responses were observed. These drugs drastically lower IgG levels by reducing the lysosomal recycling of IgG and, consequently, enhancing the elimination of pathogenic IgG. The probable upstream effect compared to complement inhibitors might explain why these drugs are much more effective.

The complement inhibitors include two monoclonal anti-C5 antibodies (Eculizumab and Ravulizumab) and their next-generation peptide-based C5 inhibitor (Zilucoplan). In our analysis, Zilucoplan was the most effective at the initiation 1w and 4w time points, while Eculizumab achieved the most efficacy at the maximized response time point. The fastest response for Zilucoplan could be possibly attributed to its once-daily injection (Eculizumab once weekly, Ravulizumab once every 2 weeks) and a dual mechanism of action (30). Additionally, Eculizumab has been proven to be an effective rescue therapy in refractory myasthenic crisis (41). The most improvement in Eculizumab could be attributed to its longest treatment period (26 weeks), compared to 12 weeks for Zilucoplan (13).

Notably, all enrolled targeted drugs did not show significant improvement compared to placebo 4 weeks post the last does, indicating effectiveness is up to maintenance treatment. Although complements or autoreactive antibodies may have been effectively purged from the blood by these drugs, autoreactive B/plasma cells and the imbalanced immune network are still unaffected. The cycle design used in most phase III MG trials may cause an unfavorable wax and wane effect for patients. Long-term positive efficacy for sustained complement usage was reported in Eculizumab and Ravulizumab (42, 43). Recently released data indicated that maintenance regimen of efgartigimod, the Q2W dosing at 10 mg/kg after one cycle continues to ensure stable symptom control, with approximately 45% of patients achieving MSE (44). Additionally, there is an ongoing maintenance trial for Batoclimab, with doses of 340 mg QW or Q2W for 52 weeks (45).

B-cell inhibitors such as Rituximab and Belimumab are postulated to take longer to take effect (e.g., Rituximab 3–6 months), as their targets are located upstream (46). In this study, Rituximab (against CD20) and Belimumab (against B-lymphocyte stimulator (BAFF)) exhibited minor differences compared to the placebo effect. Both CD20 and BAFF are involved in the survival and differentiation of B cells. Retrospective observational studies indicate that Rituximab is more effective in MuSK+ than AChR+ MG, hence the predominantly AChR+ MG composition (77.1–100%) in the current study might mask its real effect (25, 47). Interestingly, several randomized clinical trials of Rituximab in systemic lupus erythematosus had also failed, but success was achieved with Belimumab. This implies the unclear and complex B-cell-relevant mechanisms in autoimmune diseases (48). The finding of B regulatory cells (Bregs) also indicates that not all B cells are pathogenic in MG (49); hence more precise targeted therapies are needed to treat specific B-cell subgroups.

This study shows that drugs of different targets have varying values in the treatment of MG. FcRn antagonists and C5 inhibitors act quickly and significantly improve symptoms, categorizing them as fast-acting treatments similar to PLEX, IVIg, and immunoadsorption. They can be used during the induction phase of MG treatment to rapidly alleviate symptoms. Moreover, increasing evidence suggests that maintaining treatment with FcRn and C5 inhibitors may provide long-term benefits for patients (43, 44, 50). Although B-cell inhibitors did not show positive result in this study, its relatively slow onset of action and accumulating real-world evidence might support its use for maintenance therapy. Future practice could explore combinations of these biological targeted therapies, such as C5 inhibitors combined with B-cell inhibitors or FcRn antagonists combined with the C5 inhibitor (Zilucoplan) (51).

This study has several limitations. Firstly, the subjects in enrolled studies were inconsistent, as Eculizumab and Rituximab were tested on refractory and newly-onset generalized MG patients, respectively. Potential biases in study selection and data extraction may lead to misleading results. Secondly, the variance in regimens and administration routes caused biases in standardized comparisons, despite different time points being taken into account in this study. The time point selection itself can impair the generalizability of the findings. Thirdly, the immunosuppressants used in conjunction across the trials also varied, which indefinitely influenced the primary outcome of the study.

## 5 Conclusion

Within the regimen design of each trial, FcRn inhibitors (represented by Efgartigimod, Rozanolixizumab, and Batoclimab) exhibited the most effective responses in 4-week initial and maximized response in MG compared to complement and B-cell inhibitor drugs. Future research regarding long-term outcomes and real-world effectiveness of these treatments is needed to verify their efficacy.

## Data Availability

The original contributions presented in the study are included in the article/Supplementary material, further inquiries can be directed to the corresponding authors.

## Footnotes

^1^https://www.thunderheadeng.com/digxy

^2^https://www.riskofbias.info/welcome/rob-2-0-tool
