# Supplementary material for: Initiation response, maximized therapeutic efficacy, and post-treatment effects of biological targeted therapies in myasthenia gravis: a systematic review and network meta-analysis
Source: Front Neurol. 2024 Oct 28;15:1479685. doi: 10.3389/fneur.2024.1479685 (PMC11551044; doi:10.3389/fneur.2024.1479685)
Supplement: Supplementary file 1 [file Table_1.DOCX]

Supplementary Material

# Supplementary Figures and Tables

## Supplementary Figures


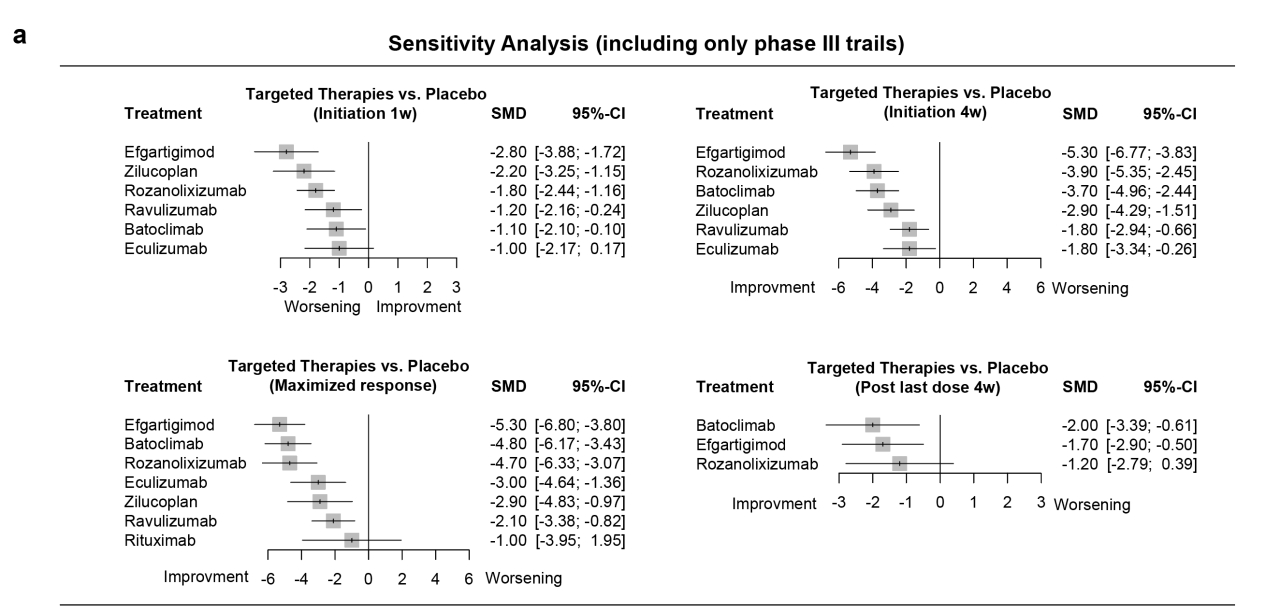


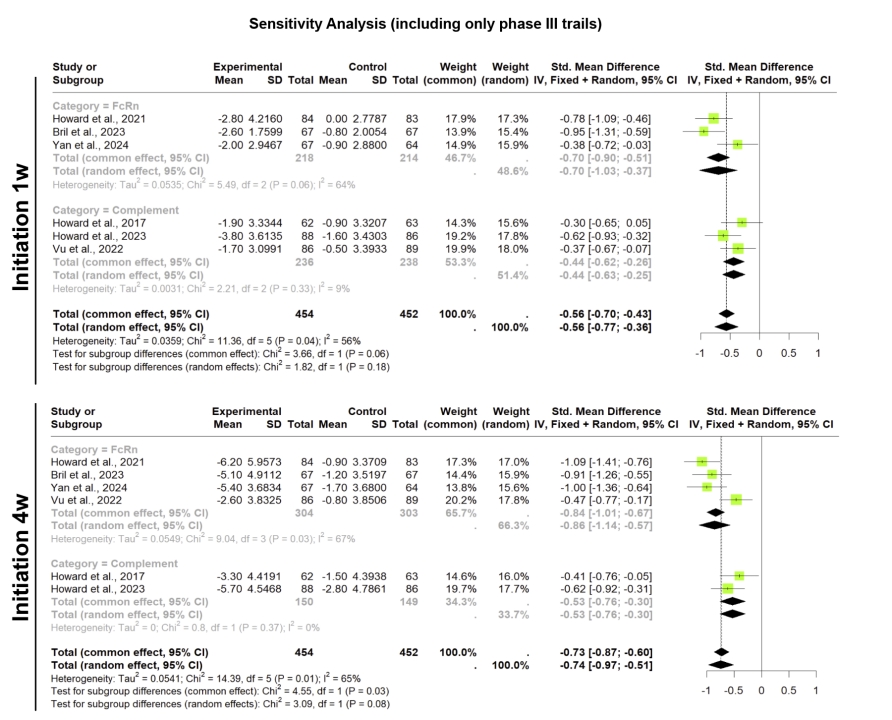

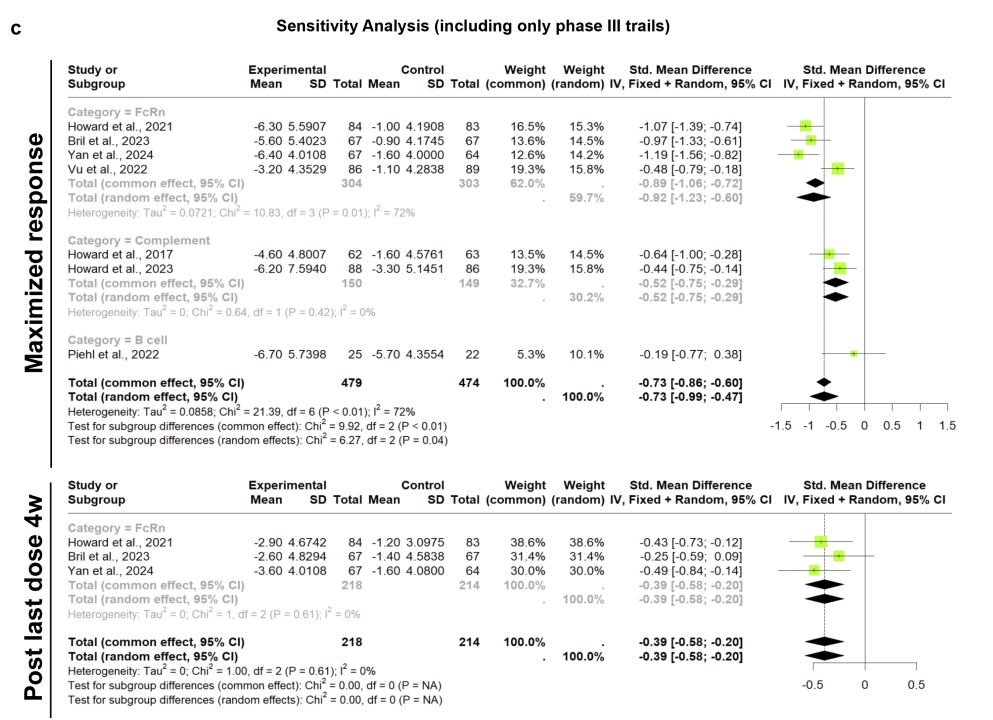


**Supplementary Figure 1. Sensitivity analysis including only phase III trial data.** Consistent results show that neonatal Fc receptor (FcRn) inhibitors are more effective than complement and B-cell inhibitors in terms of initiation 4w, maximized response, and post-treatment 4w points.
